# Observations on Phagedæna Gangrænosa

**Published:** 1818-05

**Authors:** 


### PART II.

### COMPREHENSIVE ANALYTICAL REVIEW OF MEDICAL LITERATURE.

"Tros, tyriusve, nobis nullo discrimine agetur."

*Observations on Phagedæna Gangrænosa.* By H. Home Blackadder, Surgeon. One Vol. 8vo. Edinburgh, 1818. pp. 180.

The campaigns in the Spanish peninsula, and in Belgium, have afforded numerous opportunities to our Army Surgeons for the improvement of military surgery: nor have they been inattentive or deficient in that ardour and industry so necessary towards attaining this end. As a proof of this, it will be sufficient to mention the works of Guthrie, Hennen, and, "though last not least," the volume before us.

The subject of Mr. Blackadder's observations, although less attracting to many than that of the more daring parts of operative surgery, is not less unimportant, valuable, and useful; and great praise is due to him for the improvement he suggested in the treatment of so virulent, so destructive a disease.

The work is divided into two parts. In the first we have the history and treatment of Gangrenous Phagedæna; in the second, an investigation into its history, as found in the writings of the ancients and moderns. It is to the first, or practical part, we must principally confine our attention.

Part I. By the term Phagedæna Gangrænosa is designated that disease we call hospital gangrene; the French, "la gangrene humide de hôpitaux;" and various authors, "the putrid," "scorbutic," "contagious," gangrenous ulcer," "hospital sore," "pourriture d'hôpital," &c. As this affection is a disease "sui generis," the adoption of a new name is no less requisite than allowable, and it is presumed the one proposed above will be found sufficiently correct for every useful purpose.

This disease seldom falls under the observation of civil practitioners; it occurs most commonly amongst the wounded after an engagement. When it supervenes upon a wound, its progress to a fatal termination is rapid; all structures suffer from it. It was at Passage, in the province of Biscay, in Spain, where Mr. Blackadder was appointed resident officer of a division hospital, that he first became acquainted with this disease. Three stout and healthy young men were admitted with simple flesh wounds of the lower extremities; on removing the dressings, the sores had a peculiar appearance, and in twenty-four hours got much worse. Our author was informed of the nature of their disease by Mr. Baxter, deputy inspector. A solution of nitrate of potass dissolved in vinegar superseded the use of emollients; the patients and their bedding, &c. were removed, and the disease disappeared from the hospital. Three weeks after this, four other patients were admitted with ph. gangrænosa, and in the space of a fortnight all the wards were affected. Every exertion was made, every known means tried, but nothing proved effectual in arresting or mitigating it. It was evident, therefore, that the only way to obtain these ends, was carefully and minutely to watch its progress through the different stages, and the effects of the different remedies. Mr. B. thus became satisfied of the accuracy of the following particulars:—1. There was morbid action in the sore previous to constitutional affection. 2. In several the constitution was not affected until some time after the local affection. 3. The lymphatics in the groin were often in a state of irritation before constitutional derangement was manifest. 4. In many the constitutional affection was contemporary with the second or inflammatory stage. 5. All structures were affected. 6. The disease was often confined to one sore, although the patient had many in its vicinity.

As he was of opinion that any thing which effectually destroyed the diseased action in the sore would effect the greater part of a cure, our author proposed the actual cautery; but its employment was considered inexpedient. The oxyde of arsenic then occurred to his mind; but he could procure it in no other form than Fowler's solution. This was diluted with an equal quantity of water, and the sores kept constantly wet with it by means of lint dipped therein. It occasioned considerable smarting at first, but the next morning the favourable alteration in the countenances of the patients was striking; the sores were dried up, covered with a dark insensible slough, and the pain entirely gone. Thus was the disease completely arrested, and with suitable topical applications the wounds soon got well, without any attention to the constitutional affection, which disappeared of itself.

"From this period (says Mr. Blackadder) I never saw an instance in which this method of cure failed of success when timely and properly employed."

It was adopted in the hospitals of Belgium with the same happy results.

Chap. 2. *History and Cure.*—The symptoms of phagedæna gangrænosa have been divided into local, or primary; and constitutional, or secondary.

*Local Symptoms.* Though in every instance these possess a certain general character which is common to all, they are varied by circumstances depending upon the nature of the previous injury, texture of the part affected, climate, season of the year, constitution of the patient. The peculiar expression and odour of the sore admit not of description. When it attacks a part denuded of cuticle, a small vesicle, filled with a watery or bloody fluid, is seen at the edge of the sore. If this be not ruptured, in a day or two it appears like an ash-coloured slough; if ruptured, of a dirty brownish colour. The accompanying pain resembles the stinging of a gnat. When the slough is formed, it spreads until it occupies the whole surface of the original wound, and increases in thickness daily. When the formation of the slough has been interrupted, the "stinging" becomes acute, phagedænic ulceration spreads rapidly, and in a few hours makes a considerable excavation, whilst the adjacent parts continue healthy. The edges of this cavity are well defined; it is filled with a thick glutinous matter, on removing which a fine, extremely sensible granular texture presents, very apt to bleed when touched. The sore goes on enlarging, the pain becomes more violent, the lymphatics are irritated, the discharge is copious and yellow or black; the neighbouring parts are painful, tumefied, indurated, and inflamed; the thick putrid looking slough is more and more moist, offensive matter exudes at its edges, it begins to separate, and at last is thrown off; but only to prepare the way for a repetition of the same destructive processes.

When this disease supervenes upon an old sore, where much new flesh has been formed, it is generally detected in the form of a small dark coloured spot at the edge.

Its farther progress is nearly the same as has been described, except that the phagedenic ulceration proceeds slower in the new flesh.

When it appears in recent gun-shot wounds, the discharge is found to lessen and become sanious, the sore dry and rigid, its edges elevated and sharpened, and the patient complains of the stinging pain. The surface of the wound soon assumes a livid or purple colour; it appears as if covered with a fine pellicle, which gradually increases in thickness, assuming the form of a black spongy slough. The edges are jagged, irritable, red,—their inner surface has numerous small elevated angry points, the characteristic marks of this disease.

When phagedenic gangrene attacks a recent wound, as the face of a stump, it sometimes occupies the whole surface at once; at others, it appears near the lips; but under whatever form it is first evident, its progress is, cæteris paribus, the same in nearly all cases. The attendant, local inflammation, is more or less in degree, according to the state of constitution.—In the plethoric, the sore is attacked generally from the seventh to the fourteenth day; the slough then softens to a pulp, matter oozes out at its edges, and daily increases in quantity. The inflammation subsides, and the slough is detached, leaving the subjacent muscles, bones, &c. fairly exposed: it sometimes, however, terminates in sphacelus.—In debilitated habits, the slough is longer retained, the discharge more copious, and the ulceration burrows under the integuments, which fall into a state of sphacelus. After it is detached, it sometimes, though rarely, happened that the sore gradually appeared healthy, granulated, and new skin begun to form, and notwithstanding this process was frequently interrupted by slight recurrences of ulceration, the cure has been effected by nature. It generally happens, however, that when the muscles are exposed, they are gradually dissected of cellular membrane, and left covered with an offensive greasy-looking matter, possibly the cellular and fatty substance in a state of decomposition. If the body of a muscle has been wounded, previous to the accession of the disease, it swells to a great size, and quickly assumes the appearance of a large coagulum of blood, being altogether deprived of irritability. When it has not been wounded, but has become inflamed, it is generally pale, appears as if distended with fluid, and occasionally acquires a surprising bulk. When no inflammation has supervened, the muscles become of a pale brick colour, are daily more and more wasted, and the patient loses all power over them. During the progress of the disease in the muscular system, the phagedenic ulceration continues to undermine the integuments; which, being deprived of nourishment, are seized with gangrene, and, from time to time, large sloughs are thrown off, until this unnatural dissection extends so far as to render the animal powers no longer capable of supporting its ravages. When the ligaments, or fasciæ have been exposed, the progress of the disease is generally limited, for a time, to a lateral direction, being prevented, by their texture, from extending inwards. When the bones are exposed, they soon lose their vitality, change first to a brown, and ultimately to an ebony black colour, the fœtor being almost insupportable. During the progress of the disease, bleeding from the small vessels of the sore is common,—in more advanced stages, some of the larger vessels give way, and the patient dies of hæmorrhage. When a stump is the site of the disease, and the patient plethoric, the tumefaction, pain, and heat increase rapidly, and in a few days it acquires more than twice its former size, indurated and excruciatingly painful. The patient has sometimes become delirious, and been cut off by effusion into some of the large cavities. It more commonly happens, that gangrene seizes upon the integuments and cellular substance; large sloughs are thrown off, and some of the large blood-vessels giving way, the patient perishes. For it is commonly found, that the usual modes of stopping hæmorrhage from a stump, are, in such cases, inadmissible or inefficacious.

*Constitutional Symptoms.* In no instance has Mr. B. seen these precede the local. They appear sometimes so early as the third; at others, so late as the twentieth day. The countenance is feverish; thirst; hot skin; white tongue, constipated bowels. Pulse "rather what may be termed irritated than accelerated." As was observed of the local symptoms, the disease assumes more of an inflammatory or typhoid character, as the particular causes producing these modifications have been predominant.

*Diagnosis.* Phagædena Gangrænosa is known from the simple phagedenic ulcer, by its characteristic retroverted edges. From mercurial phagedena by its history, and the peculiar nature and odour of the discharge. From some acute diseases which supervene upon a sore, as when remittent bilious fever attacks a patient after amputation, by the constitutional symptoms in these cases *preceding* the local; and the yellow suffusion generally present.

The *origin* of this disease is involved in obscurity. By some, it is attributed to the contaminated atmosphere of an hospital; whilst it is acknowledged, that the strictest attention to ventilation and cleanliness will not prevent its attack.

"Three men, who had been severely wounded, and taken prisoners, were carried to an open building, which had not been recently occupied. After having been repeatedly pillaged, they were ultimately abandoned; and the only articles left with them were a few pieces of biscuit, a canteen of water, one shirt, one pair of trowsers, a pair of shoes, and an old great coat. Thus they remained three days, when they were discovered; and some provisions and clothing being provided for them, they were put into an open boat, under the charge of a fisherman. They were exposed to a storm of wind, rain, and sleet, for nearly two days and a night, and when they got to an hospital, the wound of one of them was discovered to be affected with this disease."

It has been said to occur during the conveyance of the wounded from one station to another, when the weather has been warm and the distance considerable. Ninety cases in the hundred were evidently produced by the direct application of the morbific matter by the sponges, &c. Where this was avoided, many sores in the same ward remained unaffected.

Method of Cure.—*Indications.* 1st. To destroy the local morbid action. 2d. To regulate the reaction of the system.

At the commencement, and before the disease has arrived at that stage when the muscles, &c. come to be exposed and dissected, and a cure thereby rendered next to impossible, as the "prejudices of the times" are against the use of the actual cautery, the solution of arsenic will answer the first intention. This is to be diluted with an equal quantity of water, and constantly applied until an insensible dark-coloured dry slough occupies the whole surface of the wound, and the peculiar pain is relieved. Mr. Blackadder has heard but of one instance, where bad effects were supposed to arise from the absorption of the arsenic—and here it had been improperly applied by one unacquainted with it. To detach the slough, it must be dressed with a detergent ointment, and frequently washed with a solution of potass. When loose, it may be removed with the scissars. Lintseed poultices were too relaxing. On the detachment of the slough, the wound becomes a simple ulcer. In severe cases, amputation has been performed, but the disease soon discovered itself again, even at the first dressing, and ultimately proved fatal.

The second indication seldom requires much attention. Re-action must be moderated or supported, according to the plethoric or exhausted state of the patient. Bark does more harm than good: the primæ viæ claim attention.

### PART II.

CHAP. 1. *Authors treating of Phagædena Gangrænosa, from the beginning of the present Æra to the Sixteenth Century.*

If Celsus has not actually described this disease, he refers to a species of morbid action bearing a close resemblance to it. The description of the carbuncle, by Paulus and Rolandus, is like it in many particulars.—Avicenna refers to it. In the works of Rogerus, Brunus, Theodosius, Lanfrancus, Bertapalia, and Guido, authors of the thirteenth century, we meet with many descriptions of putrid sores, with numerous references to their various modifications, and though there be great want of precision, and even confusion, in these authors, a distinct reference to one species or modification of local morbid action, which there is reason to believe was the true phagadæna gangrænosa. We meet with it also in the writings of Alphonsus Ferrius, and Ambrose Parey.

CHAP. 2. *Authors treating of Phagadæna Gangrænosa from the beginning of the Sixteenth Century to the present.*

These are Horstius, La Motte, Wiseman, M. Ponteau, Gillespie (Med. & Phys. Journ. 1785); Blane (Observ. on the Dis. of Seamen); Adams (on Morbid Poisons); Moreau and Burdin (Mem. Soc. de Santé de Paris); Rollo (Treatise on Diabetes); John Bell (Principles of Surgery); Ballard and Harness (4th vol. Med. Phys. Journ.); Edwards (5th vol. Med. Phys. Journ.); Trotter (Med. Nautica); M. Larrey (Chirurg. Milit.); Thompson (Lectures on Inflam.); M. Delpech (Mem. sur la compl. des plaies et des Ulceres connue sous le nom de la pourriture d'Hopital.

We cannot follow our author through his detail of, and comments on, the opinions and practice of these authors. They leave us no room to doubt of his classical acquirements and professional knowledge.

The following are the conclusions drawn from the preceding inquiry:—Gangrenous phagedæna was well known to the earliest writers on surgery, and its most appropriate mode of treatment understood; but in process of time, its true character was misunderstood, and the former mode of treatment fell into disuse. Our knowledge of its nature and treatment has been greatly increased during the present age. It is first a *local* disease, and afterwards exciting a reaction in the system. It is produced by specific poison, modified by circumstances. It is *contagious*, but capable of existing in combination with other diseases of a highly *infectious* nature.

"Its remote cause is still involved in great obscurity; but an effectual method of cure is no longer a desideratum."

In a postscript, Mr. Blackadder makes some observations on that part of Mr. Hennen's work, in which Hospital gangrene is described. As Mr. B. did not see this publication before his own had passed through the press, notice could not be taken of it therein. We must presume our readers to have seen the work of Mr. Hennen, and it will not, therefore, be necessary for us to point out the difference in opinion between our author and that gentleman on the disease in question.

Thus have we finished the analysis of the work before us. We recommend it to the perusal of our readers, as one which, in point of valuable information and utility, stands second to none on the subject therein treated. It is worthy of a station on the same shelf with Guthrie and Hennen.

